# Therapeutic Effects of Phytochemicals and Medicinal Herbs on Depression

**DOI:** 10.1155/2017/6596241

**Published:** 2017-04-19

**Authors:** Gihyun Lee, Hyunsu Bae

**Affiliations:** ^1^Department of Physiology, College of Korean Medicine, Kyung Hee University, 26 Kyunghee-daero, Dongdaemun-gu, Seoul 02447, Republic of Korea; ^2^Department of Research and Development, National Development Institute of Korean Medicine, 94 Hwarang-ro, Gyeongsan-si, Gyeongsangbuk-do 38540, Republic of Korea

## Abstract

*Background*. Depression is a recurrent, common, and potentially life-threatening psychiatric disease related to multiple assignable causes. Although conventional antidepressant therapy can help relieve symptoms of depression and prevent relapse of the illness, complementary therapies are required due to disadvantage of the current therapy such as adverse effects. Moreover, a number of studies have researched adjunctive therapeutic approaches to improve outcomes for depression patients.* Purpose*. One potential complementary method with conventional antidepressants involves the use of medicinal herbs and phytochemicals that provide therapeutic benefits. Studies have revealed beneficial effects of medical herbs and phytochemicals on depression and their central nervous system mechanism. Here, we summarize the current knowledge of the therapeutic benefits of phytochemicals and medicinal herbs against depression and describe their detailed mechanisms.* Sections*. There are two sections, phytochemicals against depression and medical herbs against depression, in this review.* Conclusion*. Use of phytomedicine may be an alternative option for the treatment of depression in case conventional drugs are not applicable due to their side effects, low effectiveness, or inaccessibility. However, the efficacy and safety of these phytomedicine treatments for depression have to be supported by clinical studies.

## 1. Introduction

Depression is a recurrent, chronic, and incapacitating psychiatric ailment connected to significant mortality and morbidity [1]. This life-threatening psychic disorder is one of the most crucial reasons of disability in adults. The incidence of depression is about 3–10% in general; however, it is much higher in patients with chronic disorders, ranging between 22 and 46% [2]. Although the exact cause of depression is not known, it is thought to be influenced by the complex interactions of several genetic factors and subsequent wide exposure to environmental variables throughout lifetime. Researchers have been showing that psychological, genetic, and environmental factors remarkably increase the risk of developing this illness. The risk of this disease involves trauma, stress, and viral infections [3]. Environment-gene interplays seem to forecast a person's risk for the disease better than environment or genes alone. In addition, the childhood adversity may contribute to the disease, especially if epigenetic changes are involved [4, 5]. Structural and functional brain abnormalities seem to be related with abnormal function of the hypothalamic–pituitary–adrenal axis, low levels of brain-derived neurotrophic factor, and glutamate mediated toxicity [6].

There are three major categories in the conventional pharmacological agents for the treatment of depression: the monoamine oxidase (MAO) inhibitors, the tricyclic antidepressants, and the second-generation antidepressants. MAO inhibitors are chemicals that inhibit the activity of the monoamine oxidase enzyme family. MAO inhibitors including tranylcypromine, isocarboxazid, phenelzine, and moclobemide are often used as first-line therapy [7]. The MAO inhibitors block norepinephrine and serotonin transporter, which enhances their synaptic c levels and thus increases neurotransmission [8]. Recently, tricyclic antidepressants are being increasingly replaced by new antidepressants, which have fewer adverse effects. The new second-generation antidepressants include the norepinephrine reuptake inhibitors, the selective serotonin reuptake inhibitors, and the serotonin–norepinephrine reuptake inhibitors. Despite the development of those conventional drugs, depression treatment still fails to lead clinical remission in lots of cases [9]. It is explained by the number of interconnected systems related to depression. Moreover, many patients still display intolerant or refractory responses with these drugs [10]. Indeed, use of these agents is limited by unexpected side effects and some of them show contradictive outcomes [11, 12]. Adverse effect of antidepressant drugs involves anxiety, diaphoresis, tachycardia, tremor, sedation, insomnia, serotonin syndrome, parkinsonism, postural hypotension, blurred vision, and so forth [13, 14].

Because of these, many studies have investigated alternative curative approaches that improve clinical results against depression. Phytomedicine is one of the major complementary remedies with conventional drugs. Some phytochemicals/medical herbs have been examined for depression care and there has been a material progress. We conducted an open-ended, English restricted search of MEDLINE (Pubmed) and Scopus for all available articles up until August 31, 2016, using terms pertaining to phytomedicine, phytochemical, herb, depression, and major depressive disorder. In this review, we summarize the current knowledge of the phytochemicals/herbs and how they can improve symptoms of depression and reduce its occurrence. We also provide their mechanisms of actions.

## 2. Phytochemicals against Depression

Phytochemicals derived from herbs are known to decrease the risk of some severe disorders including autoimmune and cardiovascular diseases as well as neurodegenerative diseases. Indeed, popular polyphenols such as curcumin, ferulic acid, proanthocyanidin, quercetin, and resveratrol have shown potent anti-inflammatory and antioxidant properties. These phytochemicals repeatedly have demonstrated their neuroprotective effects, strongly suggesting that they can improve the symptoms of depression. The antidepressant activity of these phytochemicals is detailed in Table 1.

### 2.1. Carvacrol

Carvacrol is a monoterpenic phenol isolated from aromatic herbs including oregano and thyme. This aromatic phytochemical has anti-inflammatory, analgesic, antiarthritic, antiallergic, anticarcinogenic, antidiabetic, cardioprotective, gastroprotective, hepatoprotective, and neuroprotective properties [15]. This monoterpenoid phenol regulates human ion channels transient receptor potential V3 and A1 causing a sensation of warmth [16]. It is also known that carvacrol can activate PPAR and suppress COX-2 mediated inflammation [17]. Dong et al. demonstrated that enzyme cytochrome P450 2A6 (CYP2A6) is the predominant drug-metabolizing enzyme involved in the metabolism of carvacrol requesting attention when carvacrol is coadministrated with other compounds mainly undergoing CYP2A6-mediated metabolism [18]. Orally administered carvacrol (12.5–50 mg/kg) induces antidepressant effects that seem to be mediated by the dopaminergic brain pathways in mice [19]. Zotti et al. showed that carvacrol administration (12.5 mg/kg, by mouth [PO] for 7 days) can raise 5-HT and dopamine ranges in the hippocampus and prefrontal cortex [20].

### 2.2. Curcumin

Curcumin is a key active constituent of* Curcuma longa*. This yellow natural phenol has been used historically in Oriental medicine; its potential medicinal properties are under investigation [21]. Curcumin oral administration exhibited low levels in tissues and plasma, rapid metabolism, and extensive rapid excretion [22]. Insolubility in water and nonabsorption are potential factors which limit the bioavailability of curcumin; therefore multiple approaches to increase curcumin bioavailability are ongoing with the use of absorption factors, a structural analogue, liposomes, or nanomaterials [23]. Antidepressant benefit of curcumin supplement has been proven in various murine models. In rats, curcumin administration (1.25–10 mg/kg, PO for 14 days) improved damage in step-down passive avoidance and behavioral abnormalities in the open field. Curcumin treatment, in addition, decreased the immobility time in the forced swimming test and completely improved the bilateral olfactory bulbectomy-induced alteration of 5-HT, noradrenaline, and dopamine in the hippocampus [24]. Kulkarni et al. demonstrated that curcumin (20 and 40 mg/kg, intraperitoneal injection [IJ]) administration increases 5-HT level in mice and the antidepressant effect of curcumin can be increased by various kinds of antidepressants including bupropion, fluoxetine, and venlafaxine when given jointly [25]. They also showed that this phytochemical can restore biochemical and behavioral changes induced by the chronic stress [26]. Wang et al. also examined that the antidepressant benefit of curcumin (10 mg/kg, PO) involves 5-HT receptors, specifically 5-HT_1A/1B_ and 5-HT_2C_ subtypes [27]. Xu et al. again showed that curcumin (10 and 20 mg/kg, PO) can reverse 5-HT_1A_ mRNA alteration in rat hippocampus [28]. Additionally, Hurley et al. demonstrated that the antidepressant action of curcumin may be related to the increase of hippocampal brain-derived neurotrophic factor closely implicated in the pathophysiology of depression [29].

### 2.3. Ferulic Acid

Ferulic acid is phytochemical that is known to have powerful antioxidant capacity [30].

This compound is derived from phenylalanine, which is converted to 4-hydroxycinnamic acid and then caffeic acid and has shown various medicinal actions including anti-inflammatory, antitumor, antidiabetic, and neuroprotective properties [31–34]. Yabe et al. found that oral administration of ferulic acid (100 and 250 mg/kg) can mitigate stress-induced abnormal behavior in mouse depression model. Moreover, they demonstrated that ferulic acid can enhance phosphorylation of CREB and brain‐derived neurotropic factor mRNA level in the hippocampus [35].

### 2.4. L-Theanine

L-Theanine is an amino acid discovered as a component of* Camellia sinensis *(green tea) in 1949. This amino acid has been demonstrated to have various therapeutic benefits including improving concentration and learning ability, enhancing antitumor activity, preventing the vascular diseases, reducing blood pressure, providing antiobesity effect, improving the immune system, and displaying neuroprotection [36]. This behaviourally and physiologically active compound is structurally similar to the excitatory neurotransmitter glutamate and, in accordance, binds to glutamate receptors, though with much lower affinity in comparison [37]. Specifically, it binds to ionotropic glutamate receptors and acts as an antagonist of the AMPA and kainate receptors and as an agonist of the NMDA receptor [38]. L-Theanine have shown effects in the central nervous system, including the potentiation of *γ*-aminobutyric acid, dopamine, and serotonin and inhibition of glutamate reuptake [39]. With potentiation of *γ*-aminobutyric acid, the brain's main inhibitory transmitter, L-theanine, may act as a mild anxiolytic. Gomez-Ramirez et al. demonstrated the effect of L-theanine on attentional process. L-Theanine influenced continuous processes accountable for maintaining attention over a period of difficult work rather than on specific moment-to-moment phase deployment processes [40]. Administration of L-theanine (1, 4, and 20 mg/kg for 10 days) exhibited an antidepressant action in mice. In addition, L-theanine treatment markedly blocked ptosis and hypothermia induced by reserpine [41]. In the open-label clinical trial, L-theanine administration (250 mg/day for 8 weeks) was safe and manifested various beneficial effects on depressive symptoms, cognitive impairments, sleep disturbance, and anxiety in patients with depression [42].

### 2.5. Proanthocyanidins

Proanthocyanidins are oligomeric and polymeric flavan-3-ols found in various plants including apple, cocoa bean, grape, and tea. Researchers have proven that these phytochemicals have extensive pharmacological properties including cardioprotective, antioxidant effect, and antinociceptive effects [43–45]. Xu et al. have demonstrated that proanthocyanidin (25 and 50 mg/kg PO for 7 days) decreases immobility time in both forced swimming and tail suspension tests in mice. In addition, proanthocyanidin treatment increased 5-HT concentrations in hypothalamus, hippocampus, and frontal cortex. Authors suggest that the antidepressant benefit of proanthocyanidin may be associated with the central monoaminergic neurotransmitter systems [46].

### 2.6. Quercetin

Quercetin is a polyphenolic flavonoid found in many fruits, vegetables, and medicinal herbs. This flavonol has been reported to inhibit the oxidation of other molecules by acting as a scavenger of free radicals that are responsible for oxidative chain reactions [47]. Quercetin is a nonspecific protein kinase enzyme inhibitor [48] but it activates estrogen receptors [49]. In preclinical studies, quercetin (20–40 mg/kg, PO) prevented depression-like behaviors resulting from hyperactivation of the hypothalamic–pituitary–adrenal (HPA) axis in mice. The effect was comparable with fluoxetine (10–20 mg/kg, IP) [50].

### 2.7. Resveratrol

Resveratrol is a type of natural phenol found in grapes and red wine. In a clinical study with oral administration of 500 mg over 13 weeks, resveratrol was measured in cerebrospinal fluid [51]. Although 70% of orally administered resveratrol is absorbed, its bioavailability is around 0.5% because of extensive hepatic glucuronidation and sulfation [52]. One way to overcome this obstacle may be buccal delivery absorbed directly via tissues on the inside of the mouth. When 1 mg of resveratrol in 50 ml 50% alcohol/water solution was retained in the mouth for 1 minute, 37 ng/ml of resveratrol was detected in plasma 2 minutes later. This concentration could be achieved with 250 mg of resveratrol taken in a pill form [53]. Its anti-inflammatory and neuroprotective effects are proven by various researchers [54–57]. Yáñez et al. showed that resveratrol inhibits 5-HT/noradrenaline uptake and MAO activity in rats [58]. Moreover, it is showed that trans-resveratrol (20–80 mg/kg) could provide an antidepressant action, with the enhanced levels of 5-HT/noradrenaline in mouse brain [59]. Recently, Yu et al. also demonstrated that this phytochemical can inhibit chronic stress-induced depression-like behaviors. Enhanced levels of 5-HT/dopamine/noradrenaline and decreased MAO activity support the idea that resveratrol interacts with the monoaminergic system for its antidepressant effect [60].

## 3. Medical Herbs against Depression

Herbal medicine is the most popular complementary therapy [61]. Depression has prominent indications for herbal medicines [62–65] and the majority of people with depression try complementary medicines [66]. Some medicinal herbs have improved the symptoms of depression in preclinical and clinical trials (Table 2). The psychopharmacological effects of these antidepressant herbs involve regulation of 5-HT/dopamine/noradrenaline reuptake, MAO inhibition, and neuroendocrine modulation [67, 68].

### 3.1. *Camellia sinensis* (Green Tea)

Leaf of* Camellia sinensis* is a source of green tea. Green tea has shown anticancer, antifibrotic properties, anti-inflammatory, and antineurodegenerative activities [69]. Recently, preclinical study demonstrated polyphenols (5, 10, and 20 mg/kg PO for 7 days) obtained from* Camellia sinensis* improved depression-like behavior and decreased serum level of corticosterone. These results suggest that green tea polyphenols can regulate the HPA axis involved in the pathology of depression [70].

### 3.2. *Crocus sativus* (Saffron)

*Crocus sativus* is best known as the spice saffron, which is produced from parts of the flower. In two randomized controlled trials using saffron (30 mg/day), patients exhibited remarkable improvement of depression over placebo on the Hamilton Rating Scale for Depression [71, 72]. They also reported equivalent effects on Hamilton Rating Scale for Depression in three independent randomized controlled trials comparing saffron to imipramine or fluoxetine [73–75]. A limited meta-analysis concluded that saffron supplementation can improve symptoms in patients with depression [76] and another review literature indicated that it helps with mild to moderate depression [77]. Authors propose that saffron's antidepressant effects potentially are due to its serotonergic, antioxidant, anti-inflammatory, neuroendocrine, and neuroprotective effects. A recent double-blind, randomized, and placebo-controlled trial conducted by Mazidi et al. showed that saffron treatment (100 mg/day PO for 12 weeks) had a significant impact in the treatment of depression with rare side effects [78].

### 3.3. *Echium amoenum* (Borage)

*Echium amoenum* is a member of the Borage family that grows in most parts of Europe and in northern parts of Iran. The flower of* Echium amoenum* is used in alternative medicine as an antifebrile, as an anti-inflammatory, as a possible cancer protective, and as well as an antidepressant agent [79]. To elucidate the effects of* Echium amoenum* against depression, Sayyah et al. conducted a preliminary randomized, double-blind clinical trial. Results revealed that aqueous extract of* Echium amoenum* (375 mg/day PO) is superior to placebo in improving the Hamilton Rating Scale for Depression at week four of the study, although the difference was not significant at week six (*p* = 0.07) [80].

### 3.4. *Hypericum perforatum* (St John's Wort)

*Hypericum perforatum* is the most famous herb that has long been used to treat depression [95]. A number of clinical studies demonstrated that* Hypericum perforatum* is clinically efficacious for depression [96–106]. A long-term follow-up study recruited 426 responders to* Hypericum perforatum* extract (3 × 300 mg/day) to be assessed for remission rates. Results showed a beneficial effect of* Hypericum perforatum* extract for prevention from relapse after recovery from acute depression while long-term maintenance and tolerability in continuation were comparable with placebo [81]. Linde et al. showed that* Hypericum perforatum* extract is better than placebo in depression patients and is as curative as standard antidepressants but with lesser adverse effects [107]. An authoritative systematic review and meta-analysis have come to almost the same conclusion [108, 109]. Standardization and quality, however, prove difficult for* Hypericum perforatum*, as extracts show diversity of the potential effects due to unequal component profiles [110] and some clinical studies fail to support the efficacy of* Hypericum perforatum* in depression [111, 112]. Moreover,* Hypericum perforatum*'s reputation has been challenged since it is inducer of the enzyme cytochrome P450 2C9 (CYP2C9), 2C19 (CYP2C19), 3A4 (CYP3A4), and p-glucoprotein and may thus accelerate the metabolism of drugs excreted or biotransformed via these pathways [13, 113]. It was uncovered to stimulate cytochrome P450 enzyme and thus reduce the plasma level of a wide range of conventional drugs including warfarin, voriconazole, verapamil, talinolol, tacrolimus, simvastatin, quazepam, oral contraceptives, omeprazole, nifedipine, midazolam, methadone, mephenytoin, ivabradine, irinotecan, indinavir, imatinib, gliclazide, fexofenadine, erythromycin, digoxin, debrisoquine, ciclosporin, chlorzoxazone, atorvastatin, amitriptyline, and alprazolam [114–116].

### 3.5. *Piper methysticum* (Kava)

Root of* Piper methysticum* has been used for a long time to make a psychoactive drink in the South Pacific region. These days its extract is widely consumed as anxiolytic medicine in the whole world, including Europe and the United States [117]. In 2009, a 3-week double-blind crossover trial involving 60 patients was conducted to examine the anxiolytic and antidepressant effects of* Piper methysticum* extract. The study found that the aqueous extract of* Piper methysticum* (16 g containing 250 mg of kavalactones/day) supplies a remarkable improvement of comorbid depression on the Montgomery–Asberg Depression Rating Scale. Importantly, the extract did not show any grave side effects or clinical hepatotoxicity suggesting its safety as a drug [82]. However, some manufacturing companies withdrew this medicinal plant because of worry about latent hepatotoxicity [118].

### 3.6. *Rhodiola rosea* (Rose Root)

*Rhodiola rosea* is a biennial flowering plant that has been used as a traditional medicine in some Asian and European countries.* Rhodiola rosea* has been referred to as a physical and mental booster [119]. In depressive model,* Rhodiola rosea* extract (1.5–6 g/kg PO) increased 5-HT level in rat hippocampus. In addition,* Rhodiola rosea* extract induced proliferation of neural stem cell, repairing the damaged neuronal cells in the hippocampus [83]. Mattioli et al. showed antidepressant activity of* Rhodiola rosea* extract (10–20 mg/kg, PO for 3 weeks) using behavioral tests in rats. Chronic administration of* Rhodiola rosea* extract strongly inhibited chronic mild stress-induced behavioral and physiological alterations [84]. van Diermen and his coworkers showed that the antidepressant effect of* Rhodiola rosea* resulted from MAO inhibition [120]. A randomized, double-blind, placebo-controlled, phase III clinical trial using* Rhodiola rosea* extract (340–680 mg/day) demonstrated a remarkable recovery in* Rhodiola rosea*-treated group compared with placebo for mild to moderate depression [85].

### 3.7. *Lavandula angustifolia* (Lavender)

*Lavandula angustifolia* is a flowering plant in the family Lamiaceae, native to the Mediterranean. The utility of lavender oil involves an antibacterial, antifungal, carminative, sedative, and antidepressant properties [121]. Hritcu et al. reported that chronic lavender oil exposure markedly inhibited depression-like behaviors in rats in forced swimming and elevated plus-maze tests [86]. Hancianu et al. reported neuroprotective benefit of lavender oil through antioxidative effects in a rat dementia model [122]. Kasper and his colleagues have reviewed the data on the effect and tolerability of lavender oil to treat anxiety disorders [123] and the clinical trials examining safety and potential for drug interactions of lavender oil as well as its anxiolytic effect and tolerability. In the clinical trials, lavender oil was devoid of adverse effects except for mild gastrointestinal symptoms. Moreover, it did not provoke withdrawal symptoms or drug interactions at doses of 80 or 160 mg daily [124]. They also investigated the anxiolytic efficacy of lavender oil administration for the treatment of anxiety-related restlessness and disturbed sleep. It confirmed the calming and anxiolytic efficacy of lavender oil through randomized and placebo-controlled trial confirms [125]. There are also several reports demonstrating positive effect of lavender in decreasing depressive symptoms. Effati-Daryani et al. studied the effect of lavender cream on depression in pregnancy and demonstrated that the cream can be used for pregnant women to reduce depression [87]. Lavender oil infusion showed several curative benefits on depression patients, essentially decreasing mean depression score [88]. Lavender aromatherapy for 4 weeks improved the Edinburgh Postnatal Depression Scale in high risk postpartum woman [89]. I.-S. Lee and G.-J. Lee also reported that aromatherapy using lavender has a beneficial effect on depression in female college students [90]. Complementary therapy of lavender oil with imipramine for the treatment of moderate depression in 45 patients led to improved and faster results. Moreover, the anticholinergic adverse effects of imipramine, including urinary retention and dry mouth, occurred less often when imipramine was administered with lavender [91].

### 3.8. *Nelumbo nucifera*

Fruit of* Nelumbo nucifera* (Nelumbinis semen) has long been used as a natural tranquilizer in Asian countries. Our lab and coworkers have revealed its psychiatric benefits and mechanism on depression. In a rat model of depression, Nelumbinis semen showed antidepressive effects via reversing a decrease in 5-HT_1A_ receptor binding [92]; interestingly, its therapeutic effect was greater than* Hypericum perforatum*—the most popular natural antidepressant today [93]. In the forced swimming test, Nelumbinis semen treatment markedly improved the start latency, rearing number, and grooming time and decreased visiting counts caused by chronic mild stress. During 28 days of administration in dogs and 13 weeks of administration in rats, Nelumbinis semen treatment-linked toxicity was not detected [94].

## 4. Conclusion

During the last century, scientific knowledge about psychoactive herbs has remarkably progressed. Now it is a common concept that there are powerful neuroprotective phytochemicals in nature. The antidepressive actions of phytochemicals and herbs seem to be associated with various mechanisms including HPA axis, monoamine neurotransmitters, and neurogenesis/neurotrophic factors mechanisms. All these actions appear to involve promotion of the neuronal cell survival and differentiation and inhibition of neuronal cell apoptosis.

As summarized here, numerous preclinical and clinical studies have revealed the therapeutic potential of phytochemicals and medicinal herbs against depression. It is worth recommending application of these phytomedicines as an alternative antidepressant for the patients who do not benefit or do face side effects from conventional drugs. In addition, these phytomedicines could be useful therapeutic agents for the people who live in places where conventional drugs cannot be supplied to or who cannot afford conventional antidepressants as phytomedicines are considerably safe and affordable. Regardless, caution should be taken when patients use phytomedicines since not every natural product is safe. In addition, positive results or safety in animal models does not guarantee a clinical efficacy or safety in humans. Moreover, many medical plants have the potential to interact with prescribed drugs like in case of* Hypericum perforatum* [126]. Several phytochemicals and medicinal herbs with in vivo and in vitro results have still not been investigated in humans. The efficacy and safety of these phytomedicine treatments for depression have to be supported by clinical studies. Besides, in vitro and in vivo studies are also needed to discover details about their antidepressive mechanisms.

## Figures and Tables

**Table 1:** Phytochemicals against depression.

Phytochemical	Dose	Study design	Effects and mechanisms	Reference
Carvacrol	12.5–50 mg/kg	Oral administration in mice	Induce antidepressant effects that seem to be dependent on an interaction with the dopaminergic brain pathways	[19]
	12.5 mg/kg	Oral administration in rats	Raise 5-HT and dopamine ranges in the hippocampus and prefrontal cortex Influence neuronal activity through modulation of neurotransmitters	[20]

Curcumin	1.25–10 mg/kg	Oral administration in rats	Reduce immobility time in the forced swimming test Reverse bilateral olfactory bulbectomy-induced hyperactivity in the open field and deficits in step-down passive avoidance	[24]
	20–40 mg/kg	Intraperitoneal injection in mice	Enhance 5-HT level	[25]
			Restore biochemical and behavioral changes induced by the chronic stress Reverse the decreased immobility period and MAO activity induced chronic stress	[26]
	10 mg/kg	Oral administration in mice	Reduce duration of immobility in forced swimming test May be associated with 5-HT_1A/1B_ and 5-HT_2C_ subtypes	[27]
	10–20 mg/kg	Oral administration in rats	Attenuate the stress-induced hippocampus 5-HT_1A_ mRNA	[28]

Ferulic acid	100–250 mg/kg	Oral administration in mice	Attenuate stress-induced behavior Increase CREB phosphorylation and brain‐derived neurotropic factor mRNA level in the hippocampus	[35]

L-Theanine	1–20 mg/kg	Oral administration in mice	Reduce immobility time in the forced swimming test and tail suspension test without ambulation in the open field test Antagonize reserpine-induced ptosis and hypothermia	[41]

Proanthocyanidin	25–50 mg/kg	Oral administration in mice	Reduce immobility period in the forced swimming test and tail suspension test Enhance 5-HT levels in hypothalamus, hypothalamus, and frontal cortex	[46]

Quercetin	20–40 mg/kg	Oral administration in mice	Prevent hyperactivation of the HPA axis	[50]

Resveratrol	20–80 mg/kg	Oral administration in mice	Decrease immobility period in the despair tests without influence on locomotor activity Enhance 5-HT and noradrenaline concentrations in the brain	[59]
	40–80 mg/kg	Oral administration in rats	Reverse less weight gain, reduce sucrose preference and deficits in the shuttle box Raise 5-HT, dopamine, and noradrenaline concentrations in brain Reduce MAO activity	[60]

**Table 2:** Medical herbs against depression.

Herbs	Dose	Study design	Effects and mechanisms	Reference
*Camellia sinensis*	5, 10, and 20 mg/kg	Oral administration in mice	Decrease immobility in the tail suspension test and forced swimming test Modulate the HPA axis	[70]

*Crocus sativus*	30 mg/day	Randomized controlled clinical trials	Improve the Hamilton Rating Scale for Depression	[71–75]
	100 mg/day	Randomized controlled clinical trials	Improve Beck Depression Inventory and Beck Anxiety Inventory Scores with rare side effects	

*Echium amoenum*	375 mg/day	Randomized controlled clinical trials	Improve the Hamilton Rating Scale for Depression at week four of a study, however this result was not maintained at week six	[80]

*Hypericum perforatum*	3 × 300 mg/day	Long-term follow-up study involving 426 patients	Prevent relapse after recovery from acute depression	[81]

*Piper methysticum*	16 g containing 250 mg of kavalactones/day	Randomized controlled trials	Improve the Montgomery–Asberg Depression Rating Scale with no serious adverse effects and no clinical hepatotoxicity	[82]

*Rhodiola rosea *	1.5–6 g/kg	Oral administration in rats	Increase hippocampus 5-HT level-induced proliferation of neural stem cell, repairing the damaged neuronal cells in hippocampus	[83]
	10–20 mg/kg	Oral administration in rats	Revert decreased sucrose intake Reduce moving behavior Minimize weight gain and dysregulation of their estrous cycle	[84]
	340–680 mg/day	Randomized controlled phase III clinical trial	Improve overall depression, together with insomnia, emotional instability, and somatization, but not self-esteem with no serious side effects	[85]

*Lavandula angustifolia*	Lavender aromatherapy	Inhalation in rats	Inhibit depression-like behaviors in forced swimming and elevated plus-maze tests Reverse spatial memory deficits	[86]
	Lavender cream	Randomized controlled clinical trials	Reduce stress, anxiety, and depression in pregnant women	[87]
	Lavender aromatherapy	Randomized controlled clinical trials	Reduce depressive symptoms Improve the Edinburgh Postnatal Depression Scale	[88–91]

*Nelumbo nucifera*	4 g/kg	Oral administration in rats	Reverse the decreased sucrose intake and the decreased 5-HT_1A_ receptor binding in brain	[92]
	2.1 g/kg	Oral administration in rats	Increase struggling time and first latency time in the forced swimming test	[93]
	0–2000 mg/kg for rats 0–4000 mg/kg for beagle dogs	Oral administration in rats or beagle dogs	Improve the start latency, rearing number, grooming time, and decreased visiting counts caused by chronic mild stress in the forced swimming test No toxicity during 28 days of administration in dogs and 13 weeks of administration in rats	[94]

## References

[1] Nemeroff C. B. (2007). The burden of severe depression: a review of diagnostic challenges and treatment alternatives. *Journal of Psychiatric Research*.

[2] Stewart W. F. S., Ricci J. A., Chee E., Hahn S. R., Morganstein D. (2003). Cost of lost productive work time among US workers with depression. *JAMA*.

[3] Berton O., Nestler E. J. (2006). New approaches to antidepressant drug discovery: beyond monoamines. *Nature Reviews Neuroscience*.

[4] Martinowich K., Lu B. (2008). Interaction between BDNF and serotonin: role in mood disorders. *Neuropsychopharmacology*.

[5] Mitchelmore C., Gede L. (2014). Brain derived neurotrophic factor: epigenetic regulation in psychiatric disorders. *Brain Research*.

[6] Rot M. A. H., Mathew S. J., Charney D. S. (2009). Neurobiological mechanisms in major depressive disorder. *Canadian Medical Association Journal*.

[7] Shulman K. I., Herrmann N., Walker S. E. (2013). Current place of monoamine oxidase inhibitors in the treatment of depression. *CNS Drugs*.

[8] Gillman P. K. (2007). Tricyclic antidepressant pharmacology and therapeutic drug interactions updated. *British Journal of Pharmacology*.

[9] Nemeroff C. B. (2007). Prevalence and management of treatment-resistant depression. *Journal of Clinical Psychiatry*.

[10] Trivedi M. H., Fava M., Wisniewski S. R. (2006). Medication augmentation after the failure of SSRIs for depression. *The New England Journal of Medicine*.

[11] Baker C. B., Johnsrud M. T., Crismon M. L., Rosenheck R. A., Woods S. W. (2003). Quantitative analysis of sponsorship bias in economic studies of antidepressants. *British Journal of Psychiatry*.

[12] Meijer W. E. E., Heerdink E. R., Nolen W. A., Herings R. M. C., Leufkens H. G. M., Egberts A. C. G. (2004). Association of risk of abnormal bleeding with degree of serotonin reuptake inhibition by antidepressants. *Archives of Internal Medicine*.

[13] Linde K. (2009). St. John's wort—an overview. *Forschende Komplementarmedizin*.

[14] Khawam E. A., Laurencic G., Malone D. A. (2006). Side effects of antidepressants: an overview. *Cleveland Clinic Journal of Medicine*.

[15] Friedman M. (2014). Chemistry and multibeneficial bioactivities of carvacrol (4-isopropyl-2-methylphenol), a component of essential oils produced by aromatic plants and spices. *Journal of Agricultural and Food Chemistry*.

[16] Xu H., Delling M., Jun J. C., Clapham D. E. (2006). Oregano, thyme and clove-derived flavors and skin sensitizers activate specific TRP channels. *Nature Neuroscience*.

[17] Hotta M., Nakata R., Katsukawa M., Hori K., Takahashi S., Inoue H. (2010). Carvacrol, a component of thyme oil, activates PPAR*α* and *γ* and suppresses COX-2 expression. *Journal of Lipid Research*.

[18] Dong R.-H., Fang Z.-Z. E., Zhu L.-L. (2012). Identification of CYP isoforms involved in the metabolism of thymol and carvacrol in human liver microsomes (HLMs). *Pharmazie*.

[19] Melo F. H. C., Moura B. A., de Sousa D. P. (2011). Antidepressant-like effect of carvacrol (5-Isopropyl-2-methylphenol) in mice: involvement of dopaminergic system. *Fundamental and Clinical Pharmacology*.

[20] Zotti M., Colaianna M., Morgese M. G. (2013). Carvacrol: from ancient flavoring to neuromodulatory agent. *Molecules*.

[21] Wilken R., Veena M. S., Wang M. B., Srivatsan E. S. (2011). Curcumin: a review of anti-cancer properties and therapeutic activity in head and neck squamous cell carcinoma. *Molecular Cancer*.

[22] Devassy J. G., Nwachukwu I. D., Jones P. J. H. (2015). Curcumin and cancer: barriers to obtaining a health claim. *Nutrition Reviews*.

[23] Yallapu M. M., Jaggi M., Chauhan S. C. (2012). Curcumin nanoformulations: a future nanomedicine for cancer. *Drug Discovery Today*.

[24] Xu Y., Ku B.-S., Yao H.-Y. (2005). Antidepressant effects of curcumin in the forced swim test and olfactory bulbectomy models of depression in rats. *Pharmacology Biochemistry and Behavior*.

[25] Kulkarni S. K., Bhutani M. K., Bishnoi M. (2008). Antidepressant activity of curcumin: involvement of serotonin and dopamine system. *Psychopharmacology*.

[26] Bhutani M. K., Bishnoi M., Kulkarni S. K. (2009). Anti-depressant like effect of curcumin and its combination with piperine in unpredictable chronic stress-induced behavioral, biochemical and neurochemical changes. *Pharmacology Biochemistry and Behavior*.

[27] Wang R., Xu Y., Wu H.-L. (2008). The antidepressant effects of curcumin in the forced swimming test involve 5-HT1 and 5-HT2 receptors. *European Journal of Pharmacology*.

[28] Xu Y., Ku B., Cui L. (2007). Curcumin reverses impaired hippocampal neurogenesis and increases serotonin receptor 1A mRNA and brain-derived neurotrophic factor expression in chronically stressed rats. *Brain Research*.

[29] Hurley L. L., Akinfiresoye L., Nwulia E., Kamiya A., Kulkarni A. A., Tizabi Y. (2013). Antidepressant-like effects of curcumin in WKY rat model of depression is associated with an increase in hippocampal BDNF. *Behavioural Brain Research*.

[30] Srinivasan M., Sudheer A. R., Menon V. P. (2007). Ferulic acid: therapeutic potential through its antioxidant property. *Journal of Clinical Biochemistry and Nutrition*.

[31] Kawabata K., Yamamoto T., Hara A. (2000). Modifying effects of ferulic acid on azoxymethane-induced colon carcinogenesis in F344 rats. *Cancer Letters*.

[32] Balasubashini M. S., Rukkumani R., Viswanathan P., Menon V. P. (2004). Ferulic acid alleviates lipid peroxidation in diabetic rats. *Phytotherapy Research*.

[33] Sultana R., Ravagna A., Mohmmad-Abdul H., Calabrese V., Butterfield D. A. (2005). Ferulic acid ethyl ester protects neurons against amyloid *β*-peptide(1–42)-induced oxidative stress and neurotoxicity: relationship to antioxidant activity. *Journal of Neurochemistry*.

[34] Yogeeta S. K., Hanumantra R. B. R., Gnanapragasam A., Senthilkumar S., Subhashini R., Devaki T. (2006). Attenuation of abnormalities in the lipid metabolism during experimental myocardial infarction induced by isoproterenol in rats: beneficial effect of ferulic acid and ascorbic acid. *Basic and Clinical Pharmacology and Toxicology*.

[35] Yabe T., Hirahara H., Harada N. (2010). Ferulic acid induces neural progenitor cell proliferation in vitro and in vivo. *Neuroscience*.

[36] Mu W., Zhang T., Jiang B. (2015). An overview of biological production of L-theanine. *Biotechnology Advances*.

[37] Nathan P. J., Lu K., Gray M., Oliver C. (2006). The neuropharmacology of L-theanine(N-ethyl-L-glutamine): a possible neuroprotective and cognitive enhancing agent. *Journal of Herbal Pharmacotherapy*.

[38] Kakuda T., Nozawa A., Sugimoto A., Niino H. (2002). Inhibition by theanine of binding of [3H]AMPA, [3H]kainate, and [3H]MDL 105,519 to glutamate receptors. *Bioscience, Biotechnology and Biochemistry*.

[39] Lu K., Gray M. A., Oliver C. (2004). The acute effects of L-theanine in comparison with alprazolam on anticipatory anxiety in humans. *Human Psychopharmacology*.

[40] Gomez-Ramirez M., Kelly S. P., Montesi J. L., Foxe J. J. (2009). The effects of L-theanine on alpha-band oscillatory brain activity during a visuo-spatial attention task. *Brain Topography*.

[41] Yin C., Gou L., Liu Y. (2011). Antidepressant-like effects of L-theanine in the forced swim and tail suspension tests in mice. *Phytotherapy Research*.

[42] Hidese S., Ota M., Wakabayashi C. (2017). Effects of chronic l-theanine administration in patients with major depressive disorder: an open-label study. *Acta Neuropsychiatrica*.

[43] Preuss H. G., Wallerstedt D., Talpur N. (2000). Effects of niacin-bound chromium and grape seed proanthocyanidin extract on the lipid profile of hypercholesterolemic subjects: a pilot study. *Journal of Medicine*.

[44] Uchida S., Hirai K., Hatanaka J., Hanato J., Umegaki K., Yamada S. (2008). Antinociceptive effects of St. John's wort, Harpagophytum procumbens extract and grape seed proanthocyanidins extract in mice. *Biological and Pharmaceutical Bulletin*.

[45] Sato M., Bagchi D., Tosaki A., Das D. K. (2001). Grape seed proanthocyanidin reduces cardiomyocyte apoptosis by inhibiting ischemia/reperfusion-induced activation of JNK-1 and C-JUN. *Free Radical Biology and Medicine*.

[46] Xu Y., Li S., Chen R. (2010). Antidepressant-like effect of low molecular proanthocyanidin in mice: involvement of monoaminergic system. *Pharmacology Biochemistry and Behavior*.

[47] Murakami A., Ashida H., Terao J. (2008). Multitargeted cancer prevention by quercetin. *Cancer Letters*.

[48] Russo G. L., Russo M., Spagnuolo C. (2014). Quercetin: a pleiotropic kinase inhibitor against cancer. *Cancer Treatment and Research*.

[49] Moutsatsou P. (2007). The spectrum of phytoestrogens in nature: our knowledge is expanding. *Hormones*.

[50] Bhutada P., Mundhada Y., Bansod K. (2010). Reversal by quercetin of corticotrophin releasing factor induced anxiety- and depression-like effect in mice. *Progress in Neuro-Psychopharmacology and Biological Psychiatry*.

[51] Turner R. S., Thomas R. G., Craft S. (2015). A randomized, double-blind, placebo-controlled trial of resveratrol for Alzheimer disease. *Neurology*.

[52] Walle T., Hsieh F., DeLegge M. H., Oatis J. E., Walle U. K. (2004). High absorption but very low bioavailability of oral resveratrol in humans. *Drug Metabolism and Disposition*.

[53] Asensi M., Medina I., Ortega A. (2002). Inhibition of cancer growth by resveratrol is related to its low bioavailability. *Free Radical Biology and Medicine*.

[54] Tredici G., Miloso M., Nicolini G., Galbiati S., Cavaletti G., Bertelli A. (1999). Resveratrol, MAP kinases and neuronal cells: might wine be a neuroprotectant?. *Drugs under Experimental and Clinical Research*.

[55] Feng Y., Cui Y., Gao J. L. (2016). Neuroprotective effects of resveratrol against traumatic brain injury in rats: involvement of synaptic proteins and neuronal autophagy. *Molecular Medicine Reports*.

[56] Kumar A., Naidu P. S., Seghal N., Padi S. S. V. (2007). Neuroprotective effects of resveratrol against intracerebroventricular colchicine-induced cognitive impairment and oxidative stress in rats. *Pharmacology*.

[57] Ranney A., Petro M. S. (2009). Resveratrol protects spatial learning in middle-aged C57BL/6 mice from effects of ethanol. *Behavioural Pharmacology*.

[58] Yáñez M., Fraiz N., Cano E., Orallo F. (2006). Inhibitory effects of cis- and trans-resveratrol on noradrenaline and 5-hydroxytryptamine uptake and on monoamine oxidase activity. *Biochemical and Biophysical Research Communications*.

[59] Xu Y., Wang Z., You W. (2010). Antidepressant-like effect of trans-resveratrol: involvement of serotonin and noradrenaline system. *European Neuropsychopharmacology*.

[60] Yu Y., Wang R., Chen C. (2013). Antidepressant-like effect of trans-resveratrol in chronic stress model: behavioral and neurochemical evidences. *Journal of Psychiatric Research*.

[61] Ernst E., White A. (2000). The BBC survey of complementary medicine use in the UK. *Complementary Therapies in Medicine*.

[62] Eisenberg D. M., Davis R. B., Ettner S. L. (1998). Trends in alternative medicine use in the United States, 1990–1997: results of a follow-up national survey. *Journal of the American Medical Association*.

[63] Tait E. M., Laditka S. B., Laditka J. N., Nies M. A., Racine E. F. (2012). Use of complementary and alternative medicine for physical performance, energy, immune function, and general health among older women and men in the United States. *Journal of Women and Aging*.

[64] Kales H. C., Blow F. C., Welsh D. E., Mellow A. M. (2004). Herbal products and other supplements: use by elderly veterans with depression and dementia and their caregivers. *Journal of Geriatric Psychiatry and Neurology*.

[65] Roy-Byrne P. P., Bystritsky A., Russo J., Craske M. G., Sherbourne C. D., Stein M. B. (2005). Use of herbal medicine in primary care patients with mood and anxiety disorders. *Psychosomatics*.

[66] Silvers K. M., Woolley C. C., Hedderley D. (2006). Dietary supplement use in people being treated for depression. *Asia Pacific Journal of Clinical Nutrition*.

[67] Kumar V. (2006). Potential medicinal plants for CNS disorders: an overview. *Phytotherapy Research*.

[68] Sarris J. (2007). Herbal medicines in the treatment of psychiatric disorders: a systematic review. *Phytotherapy Research*.

[69] Afzal M., Safer A. M., Menon M. (2015). Green tea polyphenols and their potential role in health and disease. *Inflammopharmacology*.

[70] Zhu W.-L., Shi H.-S., Wei Y.-M. (2012). Green tea polyphenols produce antidepressant-like effects in adult mice. *Pharmacological Research*.

[71] Moshiri E., Basti A. A., Noorbala A.-A., Jamshidi A.-H., Hesameddin Abbasi S., Akhondzadeh S. (2006). Crocus sativus L. (petal) in the treatment of mild-to-moderate depression: a double-blind, randomized and placebo-controlled trial. *Phytomedicine*.

[72] Akhondzadeh S., Tahmacebi-Pour N., Noorbala A.-A. (2005). *Crocus sativus* L. in the treatment of mild to moderate depression: a double-blind, randomized and placebo-controlled trial. *Phytotherapy Research*.

[73] Akhondzadeh Basti A., Moshiri E., Noorbala A.-A., Jamshidi A.-H., Abbasi S. H., Akhondzadeh S. (2007). Comparison of petal of Crocus sativus L. and fluoxetine in the treatment of depressed outpatients: a pilot double-blind randomized trial. *Progress in Neuro-Psychopharmacology and Biological Psychiatry*.

[74] Akhondzadeh S., Fallah-Pour H., Afkham K., Jamshidi A.-H., Khalighi-Cigaroudi F. (2004). Comparison of Crocus sativus L. and imipramine in the treatment of mild to moderate depression: a pilot double-blind randomized trial [ISRCTN45683816]. *BMC Complementary and Alternative Medicine*.

[75] Noorbala A. A., Akhondzadeh S., Tahmacebi-Pour N., Jamshidi A. H. (2005). Hydro-alcoholic extract of *Crocus sativus* L. versus fluoxetine in the treatment of mild to moderate depression: a double-blind, randomized pilot trial. *Journal of Ethnopharmacology*.

[76] Hausenblas H. A., Saha D., Dubyak P. J., Anton S. D. (2013). Saffron (Crocus sativus L.) and major depressive disorder: a meta-analysis of randomized clinical trials. *Journal of Integrative Medicine*.

[77] Lopresti A. L., Drummond P. D. (2014). Saffron (*Crocus sativus*) for depression: a systematic review of clinical studies and examination of underlying antidepressant mechanisms of action. *Human Psychopharmacology*.

[78] Mazidi M., Shemshian M., Mousavi S. H. (2016). A double-blind, randomized and placebo-controlled trial of Saffron (Crocus sativus L.) in the treatment of anxiety and depression. *Journal of Complementary and Integrative Medicine*.

[79] Abolhassani M. (2010). Antiviral activity of borage (Echium amoenum). *Archives of Medical Science*.

[80] Sayyah M., Sayyah M., Kamalinejad M. (2006). A preliminary randomized double blind clinical trial on the efficacy of aqueous extract of Echium amoenum in the treatment of mild to moderate major depression. *Progress in Neuro-Psychopharmacology and Biological Psychiatry*.

[81] Kasper S., Volz H. P., Möller H. J., Dienel A., Kieser M. (2008). Continuation and long-term maintenance treatment with Hypericum extract WS® 5570 after recovery from an acute episode of moderate depression—a double-blind, randomized, placebo controlled long-term trial. *European Neuropsychopharmacology*.

[82] Sarris J., Kavanagh D. J., Byrne G., Bone K. M., Adams J., Deed G. (2009). The Kava Anxiety Depression Spectrum Study (KADSS): a randomized, placebo-controlled crossover trial using an aqueous extract of *Piper methysticum*. *Psychopharmacology*.

[83] Chen Q. G., Zeng Y. S., Qu Z. Q. (2009). The effects of Rhodiola rosea extract on 5-HT level, cell proliferation and quantity of neurons at cerebral hippocampus of depressive rats. *Phytomedicine*.

[84] Mattioli L., Funari C., Perfumi M. (2009). Effects of *Rhodiola rosea* L. extract on behavioural and physiological alterations induced by chronic mild stress in female rats. *Journal of Psychopharmacology*.

[85] Darbinyan V., Aslanyan G., Amroyan E., Gabrielyan E., Malmström C., Panossian A. (2007). Clinical trial of Rhodiola rosea L. extract SHR-5 in the treatment of mild to moderate depression. *Nordic Journal of Psychiatry*.

[86] Hritcu L., Cioanca O., Hancianu M. (2012). Effects of lavender oil inhalation on improving scopolamine-induced spatial memory impairment in laboratory rats. *Phytomedicine*.

[87] Effati-Daryani F., Mohammad-Alizadeh-Charandabi S., Mirghafourvand M., Taghizadeh M., Mohammadi A. (2015). Effect of lavender cream with or without foot-bath on anxiety, stress and depression in pregnancy: a randomized placebo-controlled trial. *Journal of Caring Sciences*.

[88] Nikfarjam M., Parvin N., Assarzadegan N., Asghari S. (2013). The effects of lavandula angustifolia mill infusion on depression in patients using citalopram: a comparison study. *Iranian Red Crescent Medical Journal*.

[89] Conrad P., Adams C. (2012). The effects of clinical aromatherapy for anxiety and depression in the high risk postpartum woman—a pilot study. *Complementary Therapies in Clinical Practice*.

[90] Lee I.-S., Lee G.-J. (2006). Effects of lavender aromatherapy on insomnia and depression in women college students. *Taehan Kanho Hakhoe Chi*.

[91] Akhondzadeh S., Kashani L., Fotouhi A. (2003). Comparison of Lavandula angustifolia Mill. tincture and imipramine in the treatment of mild to moderate depression: a double-blind, randomized trial. *Progress in Neuro-Psychopharmacology and Biological Psychiatry*.

[92] Jang C.-G., Kang M., Cho J.-H. (2004). *Nelumbinis Semen* reverses a decrease in 5-HT1A receptor binding induced by chronic mild stress, a depression-like symptom. *Archives of Pharmacal Research*.

[93] Kang M., Shin D., Oh J.-W. (2005). The anti-depressant effect of Nelumbinis Semen on rats under chronic mild stress induced depression-like symptoms. *American Journal of Chinese Medicine*.

[94] Chung H.-S., Lee H. J., Shim I., Bae H. (2012). Assessment of anti-depressant effect of nelumbinis semen on rats under chronic mild stress and its subchronic oral toxicity in rats and beagle dogs. *BMC Complementary and Alternative Medicine*.

[95] Butterweck V. (2003). Mechanism of action of St John's wort in depression—what is known?. *CNS Drugs*.

[96] Harrer G., Sommer H. (1994). Treatment of mild/moderate depressions with Hypericum. *Phytomedicine*.

[97] Kasper S., Anghelescu I.-G., Szegedi A., Dienel A., Kieser M. (2006). Superior efficacy of St John's wort extract WS®5570 compared to placebo in patients with major depression: a randomized, double-blind, placebo-controlled, multi-center trial [ISRCTN77277298]. *BMC Medicine*.

[98] Vorbach E. U., Arnoldt K. H., Hubner W.-D. (1997). Efficacy and tolerability of St. John's wort extract LI 160 versus imipramine in patients with severe depressive episodes according to ICD-10. *Pharmacopsychiatry*.

[99] Philipp M., Kohnen R., Hiller K.-O. (1999). Hypericum extract versus imipramine or placebo in patients with moderate depression: randomised multicentre study of treatment for eight weeks. *British Medical Journal*.

[100] Woelk H. (2000). Comparison of St John's wort and imipramine for treating depression: randomised controlled trial. *British Medical Journal*.

[101] Schrader E. (2000). Equivalence of St John's wort extract (Ze 117) and fluoxetine: a randomized, controlled study in mild-moderate depression. *International Clinical Psychopharmacology*.

[102] Wheatley D. (1997). Ll 160, an extract of St. John's Wort, versus amitriptyline in mildly to moderately depressed outpatients—a controlled 6-week clinical trial. *Pharmacopsychiatry*.

[103] Volz H.-P., Laux P. (2000). Potential treatment for subthreshold and mild depression: a comparison of St. John's wort extracts and fluoxetine. *Comprehensive Psychiatry*.

[104] Vorbach E.-U., Hubner W.-D., Arnold K.-H. (1993). Effectiveness and tolerance of the hypericum extract Li-160 in comparison with imipramine—Randomized double-blind-study with 135 out-patients. *Nervenheilkunde*.

[105] Harrer G., Schmidt U., Kuhn U., Biller A. (1999). Comparison of equivalence between the St. John's wort extract LoHyp-57 and fluoxetine. *Drug Research*.

[106] Mannel M., Kuhn U., Schmidt U., Ploch M., Murck H. (2010). St. John's wort extract LI160 for the treatment of depression with atypical features—a double-blind, randomized, and placebo-controlled trial. *Journal of Psychiatric Research*.

[107] Linde K., Berner M. M., Kriston L. (2009). St John's wort for major depression. *Cochrane Database of Systematic Reviews*.

[108] Rahimi R., Nikfar S., Abdollahi M. (2009). Efficacy and tolerability of Hypericum perforatum in major depressive disorder in comparison with selective serotonin reuptake inhibitors: a meta-analysis. *Progress in Neuro-Psychopharmacology and Biological Psychiatry*.

[109] Röder C., Schaefer M., Leucht S. (2004). Meta-analysis of effectiveness and tolerability of treatment of mild to moderate depression with St. John's Wort. *Fortschritte der Neurologie Psychiatrie*.

[110] Kasper S., Caraci F., Forti B., Drago F., Aguglia E. (2010). Efficacy and tolerability of Hypericum extract for the treatment of mild to moderate depression. *European Neuropsychopharmacology*.

[111] Davidson J. R. T., Gadde K. M., Fairbank J. A. (2002). Effect of Hypericum perforatum (St John's wort) in major depressive disorder—a randomized controlled trial. *The Journal of the American Medical Association*.

[112] Shelton R. C., Keller M. B., Gelenberg A. (2001). Effectiveness of St John's wort in major depression: a randomized controlled trial. *Journal of the American Medical Association*.

[113] Borrelli F., Izzo A. A. (2009). Herb-drug interactions with St John's Wort (hypericum perforatum): an update on clinical observations. *AAPS Journal*.

[114] Markowitz J. S., Donovan J. L., DeVane C. L. (2003). Effect of St John's wort on drug metabolism by induction of cytochrome P450 3A4 enzyme. *The Journal of the American Medical Association*.

[115] Mills E., Montori V. M., Wu P., Gallicano K., Clarke M., Guyatt G. (2004). Interaction of St John's wort with conventional drugs: systematic review of clinical trials. *British Medical Journal*.

[116] Izzo A. A., Ernst E. (2009). Interactions between herbal medicines and prescribed drugs: an updated systematic review. *Drugs*.

[117] Teschke R., Gaus W., Loew D. (2003). Kava extracts: safety and risks including rare hepatotoxicity. *Phytomedicine*.

[118] Pantano F., Tittarelli R., Mannocchi G. (2016). Hepatotoxicity induced by “the 3Ks”: Kava, kratom and khat. *International Journal of Molecular Sciences*.

[119] Hung S. K., Perry R., Ernst E. (2011). The effectiveness and efficacy of Rhodiola rosea L.: a systematic review of randomized clinical trials. *Phytomedicine*.

[120] van Diermen D., Marston A., Bravo J., Reist M., Carrupt P.-A., Hostettmann K. (2009). Monoamine oxidase inhibition by *Rhodiola rosea* L. roots. *Journal of Ethnopharmacology*.

[121] Cavanagh H. M. A., Wilkinson J. M. (2002). Biological activities of lavender essential oil. *Phytotherapy Research*.

[122] Hancianu M., Cioanca O., Mihasan M., Hritcu L. (2013). Neuroprotective effects of inhaled lavender oil on scopolamine-induced dementia via anti-oxidative activities in rats. *Phytomedicine*.

[123] Kasper S., Gastpar M., Müller W. E. (2010). Efficacy and safety of silexan, a new, orally administered lavender oil preparation, in subthreshold anxiety disorder—evidence from clinical trials. *Wiener Medizinische Wochenschrift*.

[124] Kasper S. (2013). An orally administered lavandula oil preparation (Silexan) for anxiety disorder and related conditions: an evidence based review. *International Journal of Psychiatry in Clinical Practice*.

[125] Kasper S., Anghelescu I., Dienel A. (2015). Efficacy of orally administered Silexan in patients with anxiety-related restlessness and disturbed sleep—a randomized, placebo-controlled trial. *European Neuropsychopharmacology*.

[126] Tsai H.-H., Lin H.-W., Simon Pickard A., Tsai H.-Y., Mahady G. B. (2012). Evaluation of documented drug interactions and contraindications associated with herbs and dietary supplements: a systematic literature review. *International Journal of Clinical Practice*.

